# The use of bilateral blink reflexes in intraoperative monitoring of facial-trigeminal nerves in cerebello-pontine angle operations

**Published:** 2012-11

**Authors:** Alireza Jamshidi Fard, Mohsen Dalvandi, Alireza Mohammadi

**Affiliations:** ^*a*^Department of Neurosurgery, School of Medicine, Arak University of Medical Sciences, Arak, Iran.

## Abstract

**Background:**

Intraoperative monitoring (IOM) of facial nerve is routinely recommended in Cerebello-Pontine Angle (CP Angle) operations. Middle cranial nerves: V, VII, VIII are mainly involved since these nerves are sometimes separated by the tumor mass causing an inadvertent section of the facial nerve. Blink reflex could be elicited by stimulation of supraorbital branch of Trigeminal nerve which elicits EMG responses in facial muscles. Threshold, amplitude, latencies, pre-post surgery are strong predictors of postoperative facial function.

**Methods:**

In 17 cases of CP angle tumors (24-43 mm, by MRI) approached suboccipitally, we performed bilateral blink reflexes pre/intra/post surgery. The setup consisted of a Nicolet Endeavor IOM system( VIASYS Healthcare, 2005, USA) with the ability to perform several voltage/current stimulations and recordingsup to 20 Evoked Potentials and Electromyography (EMG) simultaneously. Bilateral blink reflexes were evoked by stimulation of bilateral supraorbital nerves. Stimulating pulses of 0.1 ms duration and 5-20 mA intensity were applied percutaneously at the intervals of 10-20 s. The orbicularis oculi muscle responses were recorded using surface electrodes. Early EMG responses (R1) and later reflex activities (R2) were elicited ipsi/contra laterally (R1/2-i/c). Every five successive trials were superimposed and the lowest latencies were used for comparison. Blink reflexes of each subject considered pathologic if: 1- Loss of R1-i,c to the operation side, latencies are more than 15 ms or side differences are 3 ms or more; 2- Loss of R2-i, latencies are more than 50 ms or side differences are 10 ms or more. 3- Loss of R2-c, latencies are more than 55 ms or side differences are 10 ms or more. Recordings were performed 2-3 days before operaration, intraoperative and 21 days after operation.

**Results:**

Before surgery, in 15 subjects, the amplitudes of R1-i responses were significantly lower than the R1-c. However, in 2 cases with tumor size of 39 and 43 mm, the R1-i s were absent while R2-c of opposite side of the tumor were weakly present. Intraoperative recordings were continuously jugged according to patient previous results. Post operation records of all subjects showed improved amplitudes in R1-i, R1-c and R2-c with significant reduction of latencies. Propofol or Propofol/Ketamine mixture plus narcotic is suitable to record stable reproducible blink responses. Atracurium or other non-depolarizing muscle relaxant should be avoided prior to EMG recordings.

**Conclusions:**

Direct facial nerve stimulation by surgeon is often difficult in large tumors until substantial tumor mass debulked and without knowledge of the location of the nerve it sometimes, could be too late. Saving facial nerve in CP angle operations has been widely studied but optimum predictive variables have yet to be determined. The main limitation of the study method was our inability to record the reflex during electrocautery which creates large artifacts that obliterate EMG signals at the times when the risk of thermal injury caused by cautery is high. To acquire IOM modalities, close collaboration of the anesthesiologist is necessary.

**Keywords:**

Intraoperative monitoring, Facial nerve, Blink reflex, Cerebello-Pontine angle tumors


					Volume 4, Suppl. 1 Nov 2012
Publisher: Kermanshah University of Medical Sciences
URL: http://www.jivresearch.org
Copyright:
Congress of Iranian Neurosurgeons, 14-16 November 2012, Kermanshah, Iran
Paper No. 35
The use of bilateral blink reflexes in intraoperative monitoring
of facial-trigeminal nerves in cerebello-pontine angle
operations
Alireza Jamshidi Fard a,*
, Mohsen Dalvandi a
, Alireza Mohammadi a
a
Department of Neurosurgery, School of Medicine, Arak University of Medical Sciences, Arak, Iran.
Abstract:
Background: Intraoperative monitoring (IOM) of facial nerve is routinely recommended in Cerebello-Pontine Angle (CP
Angle) operations. Middle cranial nerves: V, VII, VIII are mainly involved since these nerves are sometimes separated by
the tumor mass causing an inadvertent section of the facial nerve. Blink reflex could be elicited by stimulation of
supraorbital branch of Trigeminal nerve which elicits EMG responses in facial muscles. Threshold, amplitude, latencies, pre-
post surgery are strong predictors of postoperative facial function.
Methods: In 17 cases of CP angle tumors (24-43 mm, by MRI) approached suboccipitally, we performed bilateral blink
reflexes pre/intra/post surgery. The setup consisted of a Nicolet Endeavor IOM system( VIASYS Healthcare, 2005, USA)
with the ability to perform several voltage/current stimulations and recordingsup to 20 Evoked Potentials and
Electromyography (EMG) simultaneously. Bilateral blink reflexes were evoked by stimulation of bilateral supraorbital
nerves. Stimulating pulses of 0.1 ms duration and 5-20 mA intensity were applied percutaneously at the intervals of 10-
20 s. The orbicularis oculi muscle responses were recorded using surface electrodes. Early EMG responses (R1) and later
reflex activities (R2) were elicited ipsi/contra laterally (R1/2-i/c). Every five successive trials were superimposed and the
lowest latencies were used for comparison. Blink reflexes of each subject considered pathologic if: 1- Loss of R1-i,c to the
operation side, latencies are more than 15 ms or side differences are 3 ms or more; 2- Loss of R2-i, latencies are more
than 50 ms or side differences are 10 ms or more. 3- Loss of R2-c, latencies are more than 55 ms or side differences are
10 ms or more. Recordings were performed 2-3 days before operaration, intraoperative and 21 days after operation.
Results: Before surgery, in 15 subjects, the amplitudes of R1-i responses were significantly lower than the R1-c. However, in
2 cases with tumor size of 39 and 43 mm, the R1-i s were absent while R2-c of opposite side of the tumor were weakly
present. Intraoperative recordings were continuously jugged according to patient previous results. Post operation records of
all subjects showed improved amplitudes in R1-i, R1-c and R2-c with significant reduction of latencies. Propofol or
Propofol/Ketamine mixture plus narcotic is suitable to record stable reproducible blink responses. Atracurium or other non-
depolarizing muscle relaxant should be avoided prior to EMG recordings.
Conclusions: Direct facial nerve stimulation by surgeon is often difficult in large tumors until substantial tumor mass
debulked and without knowledge of the location of the nerve it sometimes, could be too late. Saving facial nerve in CP
angle operations has been widely studied but optimum predictive variables have yet to be determined. The main limitation
of the study method was our inability to record the reflex during electrocautery which creates large artifacts that
obliterate EMG signals at the times when the risk of thermal injury caused by cautery is high. To acquire IOM modalities,
close collaboration of the anesthesiologist is necessary.
Keywords:
Intraoperative monitoring, Facial nerve, Blink reflex, Cerebello-Pontine angle tumors
* Corresponding Author at:
Alireza Jamshidi Fard: Department of Neurosurgery, School of Medicine, Arak University of Medical Sciences, Arak, Iran. Tel: 09181616251,
Email: arjfard@yahoo.com, (Jamshidi Fard A.).

				

